# Crystal structure of *catena*-poly[[di­iodidomer­cury(II)]-μ-2,2′-di­thio­bis­(pyridine *N*-oxide)-κ^2^ *O*:*O*′]^1^

**DOI:** 10.1107/S2056989018003055

**Published:** 2018-03-02

**Authors:** Rüdiger W. Seidel, Iris M. Oppel

**Affiliations:** a Lehrstuhl für Analytische Chemie, Ruhr-Universität Bochum, Universitätstrasse 150, 44780 Bochum, Germany; b Institut für Anorganische Chemie, Rheinisch-Westfälische Technische Hochschule Aachen, Landoltweg 1, 52074 Aachen, Germany

**Keywords:** crystal structure, one-dimensional coordination polymer, 2,2′-di­thio­bis­(pyridine *N*-oxide), Hg^II^ complex, di­sulfide compound

## Abstract

The title compound, [HgI_2_(C_10_H_8_N_2_O_2_S_2_)]_*n*_, a one-dimensional coordination polymer with HgI_2_ units and 2,2′-di­thio­bis­(pyridine *N*-oxide) spacer ligands in an alternating fashion, forms helical chains running along the *b* axis in the crystal. Within a single coordination polymer strand, the axially chiral 2,2′-di­thio­bis­(pyridine *N*-oxide) ligands are homochiral, but the enanti­omeric conformation is present in adjacent strands. Within a coordination polymer strand, the iodido ligands point towards the centroids of the aromatic rings of the pyridine *N*-oxide moieties in the coordination sphere of Hg^II^. Moreover, intra-strand C—H⋯O and C—H⋯I inter­actions, and inter-strand short S⋯I and S⋯O contacts are observed.

## Abstract 2

In the title compound, HgI_2_ units are joined by 2,2′-di­thio­bis­(pyridine *N*-oxide) spacer ligands in a μ-κ^2^ *O*:*O*′ coordination mode, resulting in a one-dimensional coordination polymer extending in the [010] direction.

## Chemical context   

Research into one-dimensional coordination polymers has been an active field of research, not only due to the usually easy and straightforward synthesis, but also due to inter­esting structural features and introduction of these compounds as new materials such as coordination polymeric gels, fibres and nanostructures (Leong & Vittal, 2011 ▸). In the context of our structural studies on coordination polymers and discrete metallo­supra­molecular assemblies containing di­sulfide-based bridging (spacer) ligands (Seidel *et al.*, 2013 ▸), 2,2′-di­thio­bis­(pyridine *N*-oxide) has attracted our inter­est. Recently, we reported one-dimensional coordination polymers from 2,2′-di­thio­bis­(pyridine *N*-oxide), and Zn^II^ and Cd^II^ halides (Seidel *et al.*, 2017 ▸), which represented the first structurally characterised coordination polymers containing 2,2′-di­thio­bis­(pyridine *N*-oxide) as a spacer ligand (*i.e.* involving both pyridine *N*-oxide moieties as coordinating groups). As a continuation of this work, we herein report the crystal structure of a one-dimensional coordination polymer formed from 2,2′-di­thio­bis­(pyridine *N*-oxide) and HgI_2_.
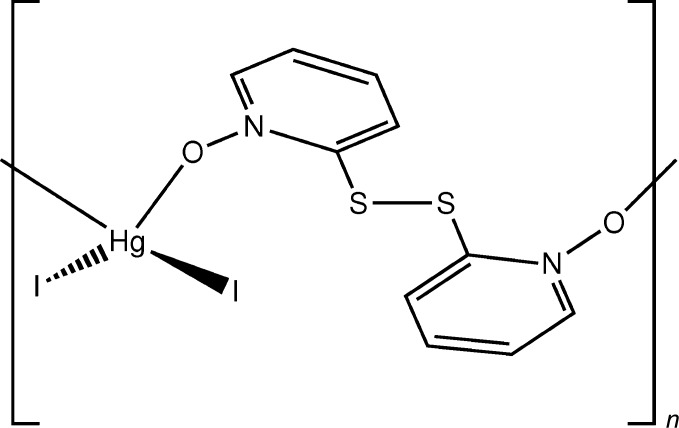


## Structural commentary   

**Figure 1:**
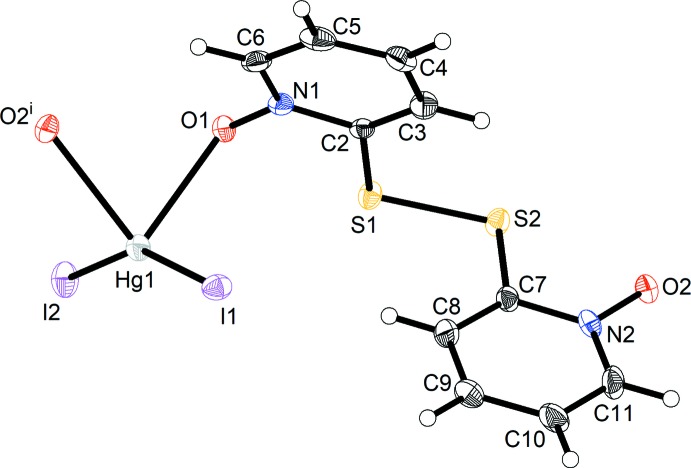
Displacement ellipsoid plot (50% probability level) of the title compound, showing the repeat unit of the coordination polymer and the coordination sphere of Hg^II^. H atoms are represented by small spheres of arbitrary radii. [Symmetry code: (i) −*x* + 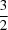, *y* − 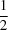, −*z* + 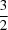.]

The title compound, (I), is a one-dimensional coordination polymer consisting of HgI_2_ units joined by 2,2′-di­thio­bis­(pyridine *N*-oxide) as bridging ligands in a μ-κ^2^ *O*:*O*′ coordination mode. Fig. 1 ▸ depicts the repeat unit of the coordination polymer and the coordination sphere of the Hg^II^ ion. The Hg^II^ ion is tetra­coordinated by two iodide ligands and two O atoms of the bridging ligands, with a coordination sphere that is best described as a severely distorted tetra­hedron or a seesaw form (Yang *et al.*, 2007 ▸). The C2—S1—S2—C7 torsion angle is 77.2 (2)°, which corresponds to the *P* form of the axially chiral *gauche* conformation of the di­sulfide-based ligand in the chosen asymmetric unit. The dihedral angle between the planes of the aromatic N1/C2–C6 and N2/C7–C11 rings is 71.8 (2)°.

**Figure 2:**
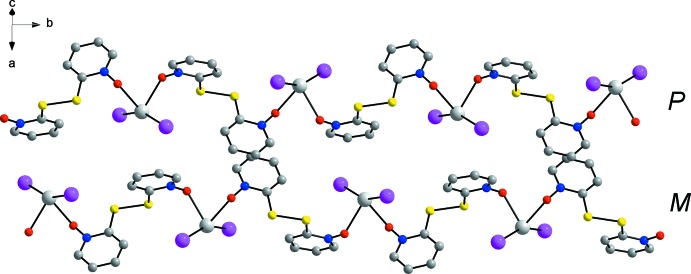
Two adjacent coordination polymer strands of the title compound, viewed along the [101] direction. *P* and *M* denote the handedness of the 2,2′-di­thio­bis­(pyridine *N*-oxide) ligand in the chains. H atoms have been omitted for clarity.

The strand of the coordination polymer propagates along a 2_1_-screw axis parallel to the *b* axis (Fig. 2 ▸). Within a single strand, 2,2′-di­thio­bis­(pyridine *N*-oxide) exhibits exclusively either the right-handed *P* or the left-handed *M* conformation, *i.e.* the bridging ligands in each coordination polymer chain are homochiral. The centrosymmetric crystal structure (space group *P*2_1_/*n*) features, however, both enanti­omeric conformations in adjacent strands, as shown in Fig. 2 ▸.

## Supra­molecular features   

In a single strand, the iodide ligands point towards the centroids of the aromatic rings of the pyridine *N*-oxide moieties in the coordination sphere of Hg^II^, but I⋯π inter­actions are not observed. The I⋯*Cg* distances are long [I1⋯*Cg*1 = 3.940 (2) Å and I2⋯*Cg*2^ii^ = 4.205 (2) Å] and the Hg—I⋯*Cg* angles are acute [Hg1—I1⋯*Cg*1 = 77.94 (3)° and Hg1—I2⋯*Cg*2^ii^ = 68.79 (3)°] [*Cg*1 and *Cg*2 are the centroids of the N1/C2–C6 and N2/C7–C11 rings, respectively; symmetry code: (ii) −*x* + 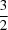, *y* − 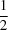, −*z* + 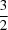]. In the chain, potentially structure-influencing C—H⋯O and C—H⋯I inter­actions can be identified (Table 1 ▸). The di­sulfide moiety is involved in two inter-strand contacts that are shorter than the sum of the van der Waals radii (Bondi, 1964 ▸); the short contacts [S1⋯I1^iii^ = 3.5983 (13) Å and S2⋯O2^iv^ = 3.263 (4) Å; symmetry codes: (iii) *x* + 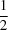, −*y* + 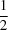, *z* + 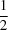; (iv) −*x* + 2, −*y* + 1, −*z* + 2], connect adjacent chains into a layer structure parallel to (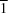
01).

## Database survey   

A search for structures containing 2,2′-di­thio­bis­(pyridine *N*-oxide) in the Cambridge Structural Database (CSD; Groom *et al.*, 2016 ▸) *via* the WebCSD inter­face (Thomas *et al.*, 2010 ▸) in February 2018 revealed the aforementioned one-dimensional coordination polymers containing Zn^II^ and Cd^II^ halide units (Seidel *et al.*, 2017 ▸). In addition, there is an Na^I^ coordination polymer, wherein the Na^I^ ions are bridged *via* only one pyridine *N*-oxide moiety of the ligand in a μ-κ^2^ *O*:*O* coordination mode (Ravindran Durai Nayagam, 2010 ▸). The crystal structures of the free, unsolvated 2,2′-di­thio­bis­(pyridine *N*-oxide) (CSD refcode RIRPEN; Bodige *et al.*, 1997 ▸) and some cocrystals (Bodige *et al.*, 1997 ▸; Bond & Jones, 2000*a* ▸,*b* ▸) have also been reported.

The isomorphous series of one-dimensional Zn^II^ coordination polymers, [Zn*X*_2_(C_10_H_8_N_2_O_2_S_2_)]_*n*_ [*X* = Cl, Br, I; C_10_H_8_N_2_O_2_S_2_ is 2,2′-di­thio­bis­(pyridine *N*-oxide)] (Seidel *et al.*, 2017 ▸) and (I) are topologically related but not isostructural. The Hg—I and Hg—O bond lengths in (I) are longer by *ca* 0.09 and 0.48 Å, respectively, than the corresponding Zn—I and Zn—O bond lengths in [ZnI_2_(C_10_H_8_N_2_O_2_S_2_)]_*n*_ (CSD refcode XAMNUX; Seidel *et al.*, 2017 ▸). The deviations of the I—Hg—I and O—Hg—O angles from the ideal tetra­hedral angle of 109.5° are considerably larger in (I) than those of the corresponding I—Zn—I and O—Zn—O angles in XAMNUX. In (2,2′-bi­pyridine *N*,*N*′-dioxide)di­iodido­mercury(II) (CSD refcode FAYKEW; Tedmann *et al.*, 2005 ▸), so far the only Hg^II^ complex with two pyridine *N*-oxide and two iodide ligands in the CSD, the I—Hg—I angle is 158.54 (4)°, which is similar to that of 155.113 (16)° observed in (I). The C2—S1—S2—C7 torsion angle in (I) [77.2 (2)°] is markedly smaller than that in RIRPEN, which is very close to the preferred value of 90° [89.89 (9)°], indicating some torsional strain in (I).

## Synthesis and crystallization   

A solution of 20 mg (0.044 mmol) HgI_2_ in 2 ml of methanol was mixed with a solution of 12 mg (0.048 mmol) 2,2′-di­thio­bis­(pyridine *N*-oxide) (Acros Organics) in 8 ml of methanol. The reaction mixture was left at room temperature and the solvent was allowed to evaporate slowly. Colourless crystals of (I) suitable for single-crystal X-ray analysis were obtained after *ca* four weeks.

After prolonged standing, colourless crystals of a second product appeared in the crystallization vessel, which were identified as [Hg_2_I_2_(C_5_H_4_NOS)_2_] (C_5_H_4_NOS^−^ is pyri­thio­nate) in a preliminary X-ray analysis.

## Refinement   

Crystal data, data collection and structure refinement details are summarized in Table 2 ▸. H-atom positions were calculated geometrically and refined using a riding model, with *U*_iso_(H) = 1.2*U*_eq_(C). The C—H bond lengths were set at 0.95 Å.

## Supplementary Material

Crystal structure: contains datablock(s) global, I. DOI: 10.1107/S2056989018003055/is5492sup1.cif

Structure factors: contains datablock(s) I. DOI: 10.1107/S2056989018003055/is5492Isup2.hkl

Supporting information file. DOI: 10.1107/S2056989018003055/is5492Isup3.mol

CCDC reference: 1825194

Additional supporting information:  crystallographic information; 3D view; checkCIF report

## Figures and Tables

**Table 1:** Hydrogen-bond geometry (Å, °)

*D*—H⋯*A*	*D*—H	H⋯*A*	*D*⋯*A*	*D*—H⋯*A*
C3—H3⋯I2^i^	0.95	3.00	3.776 (6)	140
C11—H11⋯O1^i^	0.95	2.59	3.268 (7)	129

Symmetry code: (i) 
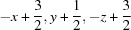
.

**Table 2:** Experimental details

Crystal data	
Chemical formula	[HgI_2_(C_10_H_8_N_2_O_2_S_2_)]
*M*_r_	706.69
Crystal system, space group	Monoclinic, *P*2_1_/*n*
Temperature (K)	108
*a*, *b*, *c* (Å)	7.4207 (3), 18.7599 (7), 11.6463 (4)
β (°)	100.887 (4)
*V* (Å^3^)	1592.13 (10)
*Z*	4
Radiation type	Mo *K*α
μ (mm^−1^)	13.81
Crystal size (mm)	0.16 × 0.10 × 0.04

Data collection	
Diffractometer	Oxford Diffraction Xcalibur2
Absorption correction	empirical (using intensity measurements) (*ShxAbs*; Spek, 2009 ▸)
*T*_min_, *T*_max_	0.614, 0.885
No. of measured, independent and observed [*I* > 2σ(*I*)] reflections	21770, 6430, 4209
*R*_int_	0.069
(sin θ/λ)_max_ (Å^−1^)	0.842

Refinement	
*R*[*F* ^2^ > 2σ(*F* ^2^)], *wR*(*F* ^2^), *S*	0.047, 0.059, 1.02
No. of reflections	6430
No. of parameters	172
H-atom treatment	H-atom parameters constrained
Δρ_max_, Δρ_min_ (e Å^−3^)	1.64, −1.39

Computer programs: *CrysAlis PRO* (Rigaku OD, 2015 ▸), *SHELXT2014* (Sheldrick, 2015*a* ▸), *SHELXL2018* (Sheldrick, 2015*b* ▸), *DIAMOND* (Brandenburg, 2016 ▸) and *enCIFer* (Allen *et al.*, 2004 ▸).

## supplementary crystallographic information

### Crystal data

**Table:**

[HgI_2_(C_10_H_8_N_2_O_2_S_2_)]	*F*(000) = 1264
*M**_r_* = 706.69	*D*_x_ = 2.948 Mg m^−^^3^
Monoclinic, *P*2_1_/*n*	Mo *K*α radiation, λ = 0.71073 Å
*a* = 7.4207 (3) Å	Cell parameters from 4297 reflections
*b* = 18.7599 (7) Å	θ = 3.6–32.1°
*c* = 11.6463 (4) Å	µ = 13.81 mm^−^^1^
β = 100.887 (4)°	*T* = 108 K
*V* = 1592.13 (10) Å^3^	Plate, colourless
*Z* = 4	0.16 × 0.10 × 0.04 mm

### Data collection

**Table:**

Oxford Diffraction Xcalibur2 diffractometer	6430 independent reflections
Radiation source: fine-focus sealed X-ray tube, Enhance (Mo) X-ray Source	4209 reflections with *I* > 2σ(*I*)
Graphite monochromator	*R*_int_ = 0.069
Detector resolution: 8.4171 pixels mm^-1^	θ_max_ = 36.8°, θ_min_ = 3.6°
ω scans	*h* = −11→10
Absorption correction: empirical (using intensity measurements) (*ShxAbs*; Spek, 2009)	*k* = −26→31
*T*_min_ = 0.614, *T*_max_ = 0.885	*l* = −15→18
21770 measured reflections	

### Refinement

**Table:**

Refinement on *F*^2^	Primary atom site location: dual
Least-squares matrix: full	Secondary atom site location: difference Fourier map
*R*[*F*^2^ > 2σ(*F*^2^)] = 0.047	Hydrogen site location: inferred from neighbouring sites
*wR*(*F*^2^) = 0.059	H-atom parameters constrained
*S* = 1.02	*w* = 1/[σ^2^(*F*_o_^2^) + (0.0045*P*)^2^] where *P* = (*F*_o_^2^ + 2*F*_c_^2^)/3
6430 reflections	(Δ/σ)_max_ = 0.001
172 parameters	Δρ_max_ = 1.64 e Å^−^^3^
0 restraints	Δρ_min_ = −1.39 e Å^−^^3^

### Special details

**Table:**

Geometry. All esds (except the esd in the dihedral angle between two l.s. planes) are estimated using the full covariance matrix. The cell esds are taken into account individually in the estimation of esds in distances, angles and torsion angles; correlations between esds in cell parameters are only used when they are defined by crystal symmetry. An approximate (isotropic) treatment of cell esds is used for estimating esds involving l.s. planes.

### Fractional atomic coordinates and isotropic or equivalent isotropic displacement parameters (Å^2^)

**Table:**

	*x*	*y*	*z*	*U*_iso_*/*U*_eq_	
Hg1	0.64620 (3)	0.14596 (2)	0.76452 (2)	0.01829 (5)	
I1	0.38759 (5)	0.22283 (2)	0.63732 (3)	0.01977 (9)	
I2	0.80134 (6)	0.07571 (2)	0.95050 (3)	0.02410 (9)	
S1	0.9109 (2)	0.33156 (8)	0.84420 (11)	0.0169 (3)	
S2	0.89904 (19)	0.43914 (8)	0.87677 (12)	0.0182 (3)	
O1	0.9177 (5)	0.2100 (2)	0.7332 (3)	0.0175 (8)	
O2	0.7700 (5)	0.5690 (2)	0.8952 (3)	0.0183 (8)	
N1	0.8795 (6)	0.2607 (3)	0.6515 (4)	0.0162 (10)	
N2	0.6271 (6)	0.5263 (2)	0.8586 (4)	0.0178 (10)	
C2	0.8761 (7)	0.3286 (3)	0.6896 (4)	0.0135 (11)	
C3	0.8382 (8)	0.3838 (3)	0.6102 (4)	0.0199 (13)	
H3	0.839772	0.431876	0.636054	0.024*	
C4	0.7978 (7)	0.3676 (3)	0.4917 (5)	0.0218 (13)	
H4	0.773405	0.404774	0.435526	0.026*	
C5	0.7931 (8)	0.2981 (3)	0.4560 (5)	0.0233 (14)	
H5	0.760912	0.287104	0.375152	0.028*	
C6	0.8351 (8)	0.2437 (3)	0.5370 (5)	0.0204 (13)	
H6	0.832653	0.195319	0.512429	0.024*	
C7	0.6611 (7)	0.4561 (3)	0.8435 (4)	0.0156 (11)	
C8	0.5189 (8)	0.4097 (3)	0.8070 (5)	0.0207 (13)	
H8	0.542666	0.360651	0.796344	0.025*	
C9	0.3408 (8)	0.4351 (3)	0.7860 (5)	0.0261 (14)	
H9	0.240770	0.403667	0.760625	0.031*	
C10	0.3099 (8)	0.5073 (3)	0.8024 (5)	0.0264 (14)	
H10	0.188027	0.525256	0.788713	0.032*	
C11	0.4533 (8)	0.5521 (3)	0.8379 (5)	0.0231 (13)	
H11	0.431858	0.601379	0.848200	0.028*	

### Atomic displacement parameters (Å^2^)

**Table:**

	*U*^11^	*U*^22^	*U*^33^	*U*^12^	*U*^13^	*U*^23^
Hg1	0.01884 (11)	0.01544 (11)	0.02109 (11)	0.00085 (10)	0.00505 (8)	0.00204 (9)
I1	0.0205 (2)	0.0230 (2)	0.01644 (18)	0.00366 (16)	0.00510 (14)	0.00259 (15)
I2	0.0291 (2)	0.0186 (2)	0.02230 (19)	0.00029 (17)	−0.00105 (16)	0.00206 (16)
S1	0.0211 (8)	0.0141 (7)	0.0146 (7)	0.0020 (5)	0.0016 (5)	0.0001 (5)
S2	0.0179 (7)	0.0155 (7)	0.0204 (7)	−0.0002 (6)	0.0017 (6)	−0.0037 (6)
O1	0.020 (2)	0.014 (2)	0.019 (2)	0.0012 (16)	0.0052 (16)	0.0031 (16)
O2	0.019 (2)	0.014 (2)	0.021 (2)	−0.0039 (16)	0.0034 (16)	−0.0005 (16)
N1	0.019 (3)	0.017 (3)	0.014 (2)	0.001 (2)	0.0071 (18)	0.0011 (19)
N2	0.022 (3)	0.014 (3)	0.018 (2)	−0.003 (2)	0.0044 (19)	0.0054 (19)
C2	0.011 (3)	0.016 (3)	0.014 (3)	−0.001 (2)	0.003 (2)	−0.002 (2)
C3	0.025 (3)	0.020 (3)	0.015 (3)	0.000 (2)	0.005 (2)	0.000 (2)
C4	0.019 (3)	0.026 (4)	0.021 (3)	0.000 (3)	0.007 (2)	0.007 (2)
C5	0.020 (3)	0.036 (4)	0.014 (3)	−0.006 (3)	0.006 (2)	−0.003 (3)
C6	0.020 (3)	0.025 (3)	0.019 (3)	−0.002 (2)	0.010 (2)	−0.007 (2)
C7	0.016 (3)	0.013 (3)	0.017 (3)	0.001 (2)	0.003 (2)	0.002 (2)
C8	0.020 (3)	0.017 (3)	0.025 (3)	−0.004 (2)	0.005 (2)	−0.004 (2)
C9	0.018 (3)	0.024 (4)	0.038 (4)	−0.008 (3)	0.010 (3)	−0.004 (3)
C10	0.016 (3)	0.024 (4)	0.041 (4)	−0.001 (3)	0.009 (3)	0.006 (3)
C11	0.023 (3)	0.012 (3)	0.033 (3)	0.002 (2)	0.004 (3)	0.004 (2)

### Geometric parameters (Å, º)

**Table:**

Hg1—O1	2.432 (4)	C3—H3	0.9500
Hg1—O2^i^	2.524 (4)	C4—C5	1.366 (8)
Hg1—I2	2.6100 (4)	C4—H4	0.9500
Hg1—I1	2.6236 (4)	C5—C6	1.385 (8)
S1—C2	1.771 (5)	C5—H5	0.9500
S1—S2	2.058 (2)	C6—H6	0.9500
S2—C7	1.764 (6)	C7—C8	1.372 (7)
O1—N1	1.336 (5)	C8—C9	1.383 (8)
O2—N2	1.333 (5)	C8—H8	0.9500
N1—C6	1.351 (6)	C9—C10	1.393 (8)
N1—C2	1.352 (7)	C9—H9	0.9500
N2—C11	1.356 (7)	C10—C11	1.357 (8)
N2—C7	1.358 (7)	C10—H10	0.9500
C2—C3	1.381 (7)	C11—H11	0.9500
C3—C4	1.389 (7)		
			
O1—Hg1—O2^i^	81.13 (12)	C5—C4—H4	120.0
O1—Hg1—I2	97.24 (8)	C3—C4—H4	120.0
O2^i^—Hg1—I2	101.01 (8)	C4—C5—C6	120.5 (5)
O1—Hg1—I1	100.48 (8)	C4—C5—H5	119.8
O2^i^—Hg1—I1	98.87 (8)	C6—C5—H5	119.8
I2—Hg1—I1	155.113 (16)	N1—C6—C5	118.6 (5)
C2—S1—S2	102.39 (19)	N1—C6—H6	120.7
C7—S2—S1	102.3 (2)	C5—C6—H6	120.7
N1—O1—Hg1	112.8 (3)	N2—C7—C8	120.3 (5)
N2—O2—Hg1^ii^	113.6 (3)	N2—C7—S2	110.4 (4)
O1—N1—C6	120.9 (5)	C8—C7—S2	129.3 (5)
O1—N1—C2	116.9 (4)	C7—C8—C9	119.4 (6)
C6—N1—C2	122.1 (5)	C7—C8—H8	120.3
O2—N2—C11	121.0 (5)	C9—C8—H8	120.3
O2—N2—C7	117.9 (5)	C8—C9—C10	119.1 (6)
C11—N2—C7	121.1 (5)	C8—C9—H9	120.4
N1—C2—C3	120.1 (5)	C10—C9—H9	120.4
N1—C2—S1	110.8 (4)	C11—C10—C9	120.3 (6)
C3—C2—S1	129.0 (4)	C11—C10—H10	119.9
C2—C3—C4	118.6 (5)	C9—C10—H10	119.9
C2—C3—H3	120.7	N2—C11—C10	119.9 (6)
C4—C3—H3	120.7	N2—C11—H11	120.1
C5—C4—C3	119.9 (5)	C10—C11—H11	120.1
			
Hg1—O1—N1—C6	−73.6 (5)	C2—N1—C6—C5	3.1 (8)
Hg1—O1—N1—C2	102.2 (4)	C4—C5—C6—N1	0.4 (9)
Hg1^ii^—O2—N2—C11	−76.5 (5)	O2—N2—C7—C8	179.4 (5)
Hg1^ii^—O2—N2—C7	103.9 (4)	C11—N2—C7—C8	−0.2 (8)
O1—N1—C2—C3	179.8 (5)	O2—N2—C7—S2	−0.6 (6)
C6—N1—C2—C3	−4.4 (8)	C11—N2—C7—S2	179.8 (4)
O1—N1—C2—S1	−3.7 (6)	S1—S2—C7—N2	−179.1 (3)
C6—N1—C2—S1	172.1 (4)	S1—S2—C7—C8	0.9 (6)
S2—S1—C2—N1	−180.0 (3)	N2—C7—C8—C9	−0.1 (8)
S2—S1—C2—C3	−3.9 (6)	S2—C7—C8—C9	179.9 (4)
N1—C2—C3—C4	2.3 (8)	C7—C8—C9—C10	0.0 (9)
S1—C2—C3—C4	−173.6 (4)	C8—C9—C10—C11	0.4 (9)
C2—C3—C4—C5	1.1 (9)	O2—N2—C11—C10	−179.0 (5)
C3—C4—C5—C6	−2.4 (9)	C7—N2—C11—C10	0.6 (8)
O1—N1—C6—C5	178.7 (5)	C9—C10—C11—N2	−0.7 (9)

Symmetry codes: (i) −*x*+3/2, *y*−1/2, −*z*+3/2; (ii) −*x*+3/2, *y*+1/2, −*z*+3/2.

### Hydrogen-bond geometry (Å, º)

**Table:**

*D*—H···*A*	*D*—H	H···*A*	*D*···*A*	*D*—H···*A*
C3—H3···I2^ii^	0.95	3.00	3.776 (6)	140
C11—H11···O1^ii^	0.95	2.59	3.268 (7)	129

Symmetry code: (ii) −*x*+3/2, *y*+1/2, −*z*+3/2.
